# Pilates-Based Training and Its Influence on Muscle Viscoelasticity and Health-Related Outcomes in Chronic Low Back Pain: A Comparative Study

**DOI:** 10.3390/healthcare14040448

**Published:** 2026-02-11

**Authors:** Onur Aydoğdu, Osman Çoban, Yağmur Tetik Aydoğdu, Azime Yıldız, Zübeyir Sarı

**Affiliations:** 1 Department of Physiotherapy and Rehabilitation, Faculty of Health Sciences, Marmara University, İstanbul 34854, Turkey; azimeyildiz99@gmail.com (A.Y.); zubeyirsari@gmail.com (Z.S.); 2 Department of Physiotherapy and Rehabilitation, Faculty of Health Sciences, İstanbul Üsküdar University, İstanbul 34662, Turkey; oscoban28@gmail.com; 3 Sureyyapasa Chest Diseases and Thoracic Surgery Training and Research Hospital, İstanbul 34844, Turkey; yagmuraydogdu@outlook.com

**Keywords:** low back pain, Pilates-based exercises, viscoelastic properties

## Abstract

Background: The viscoelastic properties of muscle tissue are important factors affecting muscle performance; they play a significant role in maintaining spinal stability, as well as muscle contraction and function. Changes in these properties can result in pain, restricted movement, or poor posture. However, there is limited information in the literature regarding the viscoelastic properties of the paraspinal muscles, such as tone and stiffness, in individuals with chronic low back pain, which is one of the most common musculoskeletal disorders. The main aim of our study was to investigate the effects of reformer Pilates exercises on muscle viscoelastic properties in individuals with chronic low back pain for 4 weeks. In addition, our secondary aim was to examine the effects of Pilates-based exercises on body anthropometric values, pain intensity, functionality and kinesiophobia levels, sleep, and quality of life in individuals with chronic low back pain and to compare these parameters with a healthy group without low back pain. Methods: The study was carried out in a private clinic center and involved a total of 52 participants: 24 healthy subjects (control group) and 28 subjects with chronic low back pain (CLBP group). Pilates-based exercises were applied 2 days a week for 8 sessions for a total of 4 weeks. Muscle viscoelastic properties, body anthropometric values, pain intensity, functional status, kinesiophobia, sleep quality, and quality of life of all cases were evaluated. Muscle viscoelastic values were measured with a portable myotonometer, MyotonPro. Results: After 4 weeks of Pilates-based training, no significant improvements were observed in the parameters of muscle tone and stiffness in both groups (*p* > 0.05). It was found that pain intensity (*p* = 0.001), sleep quality (*p* = 0.004), quality of life (*p* = 0.019), and disability level (*p* = 0.003) improved after 4 weeks of Pilates-based training in subjects with chronic low back pain. In addition, there were significant differences in the parameters of the chest, waist, hip, and thigh circumferences after 4 weeks of Pilates-based training, except for the abdomen, in both groups (*p* < 0.05). Conclusions: A period of four weeks of Pilates exercises did not lead to significant changes in the muscle viscoelastic properties of the lumbar and abdominal muscles, although performing these exercises did result in regional thinning. The efficacy of Pilates exercises in reducing pain, disability, and kinesiophobia and in improving sleep and quality of life has been demonstrated in individuals suffering from chronic low back pain.

## 1. Introduction

Chronic low back pain (CLBP) is defined as persistent low back pain for at least 3 months without any specific cause. It is one of the most common musculoskeletal disorders, and it has been stated that more than 80% of the population worldwide has/will have a history of low back pain [1]. It affects public health and also imposes a serious burden on the economy. For example, the total annual cost of low back pain in the UK is estimated to be £12 billion [2]. Chronic low back pain can often be associated with a sedentary lifestyle, obesity, or poor working conditions [3].

Muscle tone and stiffness, also known as the viscoelastic properties of muscles, are important elements that influence muscle performance. They play a key role in maintaining muscle contraction and function, as well as spinal stability [4]. Changes in muscle tone can result in pain, limited control of movement, or poor posture. High muscle tone, in particular, is thought to cause chronic pain in adults due to the pain–spasm–pain cycle [5]. Changes in the tone of the paraspinal muscles may contribute to swelling of the intervertebral disks and/or flattening of the spinal curvature, resulting in low back pain [6]. There is limited information in the literature regarding the viscoelastic properties of muscles, such as tone and stiffness, in individuals with chronic low back pain. One of these studies detected altered tone and stiffness in the lumbar myofascial region in individuals with chronic low back pain. It may be possible to manage chronic low back pain by reducing paraspinal muscle activity through rehabilitation interventions such as manual therapy or therapeutic exercises [7].

MyotonPro (Myoton AS, Tallinn, Estonia) is a portable muscle detector that quickly and non-invasively measures muscle and myofascial tissue tone and stiffness [4]. It is an accurate and reliable device for testing the viscoelastic properties of muscles and myofascial tissues in both healthy and sick individuals. The principle behind it is to apply multiple short pulses to the muscle via the test probe to create oscillations in the muscle fibers. MyotonPro enables the measurement of paraspinal myofascial tissue tone through the analysis of induced tissue oscillations [6].

The Pilates method, which is a systematic exercise approach using resistive exercises to increase muscle strength [8], is one of the therapeutic methods applied in the treatment of chronic low back pain [9]. Pilates is widely recommended by many doctors and physiotherapists to the adult and elderly population due to its positive effects on balance and fall prevention in individuals with chronic low back pain [10]. Some clinical studies have demonstrated that Pilates has a positive, low-to-moderate effect on health-related outcomes such as pain, disability, functional status, and kinesiophobia in individuals with chronic low back pain [3,11,12]. However, some other studies have shown that Pilates is not significantly different from traditional physiotherapy and rehabilitation [11]. More importantly, there is evidence that Pilates training leads to changes in muscle activation [12]. In individuals with chronic low back pain, some back and abdominal muscles such as transversus abdominis and multifidus muscles may be inhibited due to chronic pain; Pilates exercises aim to activate these muscles [10]. For this reason, there have been interesting studies examining the effects of Pilates-based exercises on the electromyographic activation of abdominal and lumbar muscles [13,14]; however, to the best of our knowledge, there is no study examining muscle viscoelastic properties such as tone and stiffness of superficial muscles in the lumbar and abdominal region after Pilates exercises in individuals with chronic low back pain.

In light of this information, the main aim of our study was to investigate the effects of reformer Pilates exercises on muscle viscoelastic properties in individuals with chronic low back pain for 4 weeks. In addition, our secondary aim was to examine the effects of Pilates-based exercises on body anthropometric values, pain intensity, functionality and kinesiophobia levels, sleep, and quality of life in individuals with chronic low back pain and to compare these parameters with a healthy group without low back pain.

Research Questions:

1. Do reformer Pilates exercises performed for four weeks have effects on muscle viscoelastic properties, body anthropometric values, pain, functionality, kinesiophobia, sleep, and quality of life in individuals with chronic low back pain?
2. Does the presence of chronic back pain have any impact on the improvement in the relevant parameters?

## 2. Materials and Methods

### 2.1. Study Design and Setting

This study was designed as a prospective and comparative trial. The study was carried out in a private clinic center in Istanbul and included a total of 52 participants: 24 healthy subjects and 28 subjects with chronic low back pain. The control group consists of healthy individuals who have not received any diagnosis and do not experience pain, particularly in the lumbar region. The CLBP group consists of individuals who have experienced non-specific pain in the lumbar region for at least three months. The only difference between the two groups was the presence of lower back pain. Both groups visited the center during the same period.

### 2.2. Inclusion and Exclusion Criteria

The inclusion criteria were to be aged between 18 and 60 years and to have a history of non-specific chronic low back pain of at least 12 weeks’ duration. In addition, subjects were considered to be eligible for inclusion if they had a sedentary lifestyle (individuals who have not been exercising regularly recently and who do not engage in moderate- to high-intensity physical activity for 150 min or more per week) and had not undergone surgery in the lumbar spine region. A physiotherapist with 15 years’ experience was responsible for the review of the eligibility assessment of subjects with chronic low back pain. Subjects were excluded if they had a history of spinal trauma, a medical diagnosis of spondylosis, spondylolisthesis, scoliosis or herniated disk with radiculopathy, a history of spinal surgery or spinal stenosis, a diagnosis of neurological and psychological conditions, cancer or pregnancy in the previous year, physiotherapy and rehabilitation in the previous year, missed the two subsequent Pilates sessions, or did not attend the assessment sessions.

### 2.3. Ethical Approval

This study was conducted in accordance with the Declaration of Helsinki. All subjects gave their signed written informed consent after being informed of the procedures to be performed. This study was approved by the Ethics Committee of Non-Interventional Clinical Research of Marmara University, Faculty of Health Sciences (protocol number: 06.07.2023/88).

### 2.4. Sample Size

The study sample size was calculated using the software G-Power version 3.1.9.3 (Heinrich Heine University Düsseldorf, Germany) and based on a study by Hyeon Ko et al. examining the effects of lumbar stabilization and lower extremity strength exercise on lumbar pain and physical function in middle-aged women with chronic back pain [15]. In this study, the muscle stiffness score as the viscoelastic properties level of those participants with low back pain was calculated for the treatment group in which lumbar stabilization and lower extremity strength exercise was applied, using pre-treatment values of 211.22 ± 26.97 and post-treatment values of 187.00 ± 24.70. With 85% power and an α error coefficient of 0.05, at least 22 cases were required per group. Despite the possibility of patients discontinuing the treatment program, the number of cases in each group was increased by 10% to ensure a minimum of 24 cases.

### 2.5. Interventions

All Pilates-based exercises were prescribed by a qualified physiotherapist with clinical experience in treating patients with low back pain. The exercise sessions were approximately 50 min in duration and were carried out by a physiotherapist working as a Pilates instructor.

The subjects were administered a reformer Pilates-based exercise program over a period of four weeks, with sessions conducted twice weekly and a minimum interval of two days between each session. All participants were given 1 introductory session by the physiotherapist to understand the basic philosophy and principles of Pilates exercises, to demonstrate correct breathing techniques, and to adapt to the Reformer machine. In addition, all participants were given a 10 min warm-up and cool-down program consisting of stretching exercises for the major muscle groups of the lower extremities and spine, including the hip, knee, lower back, and core muscles.

All strengthening exercises were performed with a two-minute rest period between each set. The intensity of the exercises was initially set at a beginner level to minimize the risk of injury to participants and was then progressively increased over time. The exercise regime consisted of eight repetitions of two sets during the first two weeks and twelve repetitions of three sets during the last two weeks (Supplementary Material S1).

### 2.6. Measurement Tools

Sociodemographic and clinical outcomes and anthropometric data were assessed by an experienced physiotherapist. All interviews were performed face-to-face. Sociodemographic information, dominant side, comorbidities, education and occupational status, medication use, and life habits were questioned.

The main outcome measurement was viscoelastic properties of the rectus abdominis and erector spinae muscles. Secondary outcome measurements were anthropometric measurements, pain intensity, sleep quality, quality of life, kinesiophobia, and disability.

Measurements of primary and secondary outcomes were performed at baseline and in the 4-week follow-up by the same assessor.

#### 2.6.1. Viscoelastic Properties—Myotonometric Assessment

Muscle viscoelastic values were measured with a portable myotonometer. Muscle stiffness and tone in the rectus abdominis and lumbar erector spinae muscles of all subjects were evaluated using Myoton Pro device (Myoton AS, Tallinn, Estonia). Myotonometry is a non-invasive, reliable, and valid method for assessing muscle viscoelastic properties of tone, stiffness, and elasticity. In a study by Yapeng Li et al., it was shown that the device is applicable in patients with chronic low back pain [16]. The mechanical impulse applied by the portable device to the skin is first transmitted to the soft tissue and then to the muscles (0.58 N for 15 ms). The impulse is recorded by the probe at the end of the device to calculate viscoelastic properties. In this study, there are 2 different muscle viscoelastic and biomechanical properties evaluated. The first one, F—frequency [Hz], represents muscle tone, while the other one, S—stiffness [N/m], represents muscle stiffness [17].

All muscle viscoelastic assessments were performed by a physiotherapist experienced in using the device. The assessor had previously used the same device many times in other studies. Before using the device, the assessor practiced palpation of the rectus abdominis and lumbar erector spinae muscles. The measurements were performed in a quiet laboratory environment at room temperature (approximately 25 °C). For muscle stiffness and tonus measurements, 5 consecutive impulses were applied to the same point in the same muscle and averaged automatically by the device. The probe of the device was applied perpendicular to the muscle stems.

For the measurement of the lumbar erector spinae muscle, the participant lay in the prone position. The participant’s arms were positioned next to the body and the head in neutral position. A few centimeters lateral to the spinous process at the L4 level was chosen as the measurement point [16].

For the rectus abdominis muscle measurement, the participant lay in the supine position. The participant’s arms were positioned at his/her side, legs in the hooked position, and head in the neutral position. Three centimeters lateral and inferior to the umbilicus was selected as the measurement point [17].

#### 2.6.2. Anthropometric Measurements

In order to determine the physical characteristics of all participants, some anthropometric evaluations such as body weight, height, and circumference measurements were performed. All anthropometric evaluations were repeated at baseline and after 4 weeks. A digital weighing scale with a precision of 0.1 kg was used for body weight measurement and recorded in kg. For height, a simple height gauge was used and recorded in cm. Body mass index was calculated by dividing body weight by the square of height and reported in kg/m^2^. Circumferences of the chest, waist, abdomen, hips, and thighs were measured using a simple tape measure and recorded in cm.

#### 2.6.3. Pain Intensity

The visual analog scale was used, which is considered to be an acceptable scale for measuring pain intensity and has a simple, free, and quick application in assessing the level of pain intensity of subjects with low back pain. The subjects were instructed to indicate a point on a line between 0 and 10 cm, with 0 representing no pain and 10 representing the maximum possible pain [18,19].

#### 2.6.4. Sleep Quality

Sleep quality was assessed using the Pittsburgh Sleep Quality Index (PSQI). The PSQI is a self-report scale that assesses sleep quality and disturbances experienced by individuals over the previous 4 weeks. The PSQI consists of seven subcomponents: subjective sleep quality, time to sleep onset, sleep duration, sleep efficiency, sleep disturbances, use of sleep medication, and daytime functioning. The total PSQI score is obtained by summing the scores of these seven items. The total score ranges from 0 to 21, with higher scores indicating poorer sleep quality. Individuals with an overall PSQI score of 5 and below are considered to have “good sleep quality”, while scores above 5 indicate “poor sleep quality”. This scale was developed by in 1989, and a study of its validity and reliability was conducted by Ağargün et al. in Turkey. The PSQI scale is widely used to assess sleep quality and related factors [20,21,22].

#### 2.6.5. Quality of Life

The health-related quality of life of all subjects was assessed using the Nottingham Health Profile (NSP). The NSP is a quality of life survey that assesses health problems perceived by the participant and the extent to which these problems interfere with activities of daily living. The survey was developed in 1981 and adapted to Turkish by Küçükdeveci et al. in 1997 [23]. The survey consists of 2 distinct sections. In this study, the results of the first section of the scale were evaluated. The first section of the survey consists of 38 items and 6 dimensions. Each question is answered with “yes” or “no”. As the score on the scale increases, the general health status deteriorates [23].

#### 2.6.6. Kinesiophobia

The level of kinesiophobia (fear of movement) was measured using the Tampa Kinesiophobia Scale. The scale was published in 1995. The Tampa Kinesiophobia Scale, developed to assess fear of movement or reinjury, is a 17-item scale. The scale includes 4-point Likert responses ranging from “I strongly disagree” to “I strongly agree”. The person scores between 17 and 68, with higher scores indicating greater kinesiophobia. The validity and reliability of the scale in Turkey were established by Tunca-Yılmaz et al. [24].

#### 2.6.7. Disability

Functional levels were measured using the Oswestry Disability Index. The ODI, defined in 1980, consists of 10 different subscales that assess pain intensity, personal care, heavy lifting, walking, sitting, standing, sleeping, sexual life, social life, travel, and pain. Each subscale is scored between 0 and 5. A high score indicates a high level of disability. The validity and reliability of the scale in Turkish were established by Yakut et al. [25].

### 2.7. Statistical Analyses

The Statistical Package for the Social Sciences (SPSS) program (IBM Corp., Armonk, NY, USA), version 11.5, was used to perform the statistical analyses for the study. All continuous variables were expressed as the mean and standard deviation (SD). Normal distribution of the data was evaluated using the Shapiro–Wilk test and frequency histograms. Skewness and kurtosis values were also calculated to confirm data normality. Wilcoxon’s signed rank test was used to compare the mean difference between the baseline and follow-up parts of the intervention within the groups, while the Mann–Whitney U Test was used to examine the mean differences between the groups. The significance level was considered as *p* < 0.05 in all tests.

## 3. Results

### 3.1. Participants

In total, 24 healthy subjects and 28 subjects with chronic low back pain participated in the study. While 18 of the subjects in the healthy group were female (75.0%), all of the subjects in the low back pain group were female. All participants in the study fully complied with the interventions, and all participants completed the process.

The age range of all the participants ranged from 18 to 58 years, with a mean of 33.90 ± 11.25 years. Of the 52 subjects who participated in the study, 30 were “bachelor” (57.7%), and the majority of the subjects had the right dominant limb (92.3%). Descriptive statistics of the participants are presented in Table 1.

### 3.2. Changes in Muscle Viscoelasticity

Table 2 indicates the pre- and post-training muscle viscoelastic properties of the rectus abdominis and erector spinae muscles. After the 4-week Pilates-based training program, no significant improvements were observed in the parameters of muscle tone or stiffness in both groups (*p* > 0.05). In addition, no statistical differences were found in terms of muscle tone or stiffness by group (*p* > 0.05).

### 3.3. Changes in Clinical Outcomes and Circumference Measurements

Table 3 reflects the effects of the 4-week Pilates-based training program on pain intensity, sleep quality, quality of life, kinesiophobia, and disability levels in both groups. It was found that pain intensity (*p* = 0.001), sleep quality (*p* = 0.004), quality of life (*p* = 0.019), and disability level (*p* = 0.003) improved after the 4-week Pilates-based training program in the CLBP group. A significant improvement in the kinesiophobia levels was shown in both groups (*p* = 0.001).

Regarding the comparison of the differences between the groups, significant differences were found for pain intensity (*p* = 0.001) and sleep quality (*p* = 0.042).

The mean values of the circumference measurements pre- and post-training are shown in Table 3. In both groups, there were significant differences in the parameters of the chest, waist, hip, and thigh circumferences after 4 weeks of Pilates-based training, except for the abdomen circumferences (*p* < 0.05). On the other hand, there was no statistically significant difference between the healthy and CLBP groups in terms of circumference measurements (*p* > 0.05).

## 4. Discussion

To the best of our knowledge, there is no research in the literature optimizing viscoelastic properties such as altered tonus and stiffness in individuals with chronic low back pain. Therefore, the main aim of this study was to examine the effects of Pilates-based exercises on the viscoelastic properties of lumbar and abdominal muscles in individuals with chronic low back pain. The main finding of this study was that the performance of Pilates-based exercises for 4 weeks did not significantly change the tone and stiffness levels of the erector spina or rectus abdominis muscles. To the best of our knowledge, there is no research examining the effects of the Pilates method on muscle tone or stiffness in any population. Similar to our study, Machado et al. reported that performing Pilates exercises for 8 weeks increased lumbar multifidus muscle activation from deep muscle groups in individuals with low back pain [14]. Similarly, Bueno Silva et al. found that the performance of Pilates exercises resulted in higher muscle activation in the upper rectus abdominis muscles compared to traditional exercises. On the other hand, no significant difference was found in the lower rectus abdominis muscles [13]. In our study, contrary to the literature, we did not examine the effects of the Pilates method on muscle activation with EMG measurements; rather, we examined viscoelastic properties such as the tone and stiffness of superficial level muscles with Myoton Pro, not deep muscles [26]. The fact that Pilates-based exercises did not significantly change muscle tone and stiffness both in the healthy control group and in individuals with chronic low back pain is primarily attributed to the short duration of the Pilates method applied. This is because, in similar published studies, Pilates-based exercises were applied for at least 6 [8], usually 8 [14,27], and sometimes even 10, weeks [28]; in our study, it was applied for almost half of this period. The main reason for choosing Pilates exercises for 4 weeks was that the continuation rate of individuals to Pilates in our region is usually 4 weeks, and the packages offered for sale in Pilates clinical centers are usually 1-month packages. As a result, we think that duration may be an important factor and muscle viscoelastic properties will not change significantly with 4 weeks of Pilates exercises. Similar to our study, it was found that the thoracic spine self-mobilization technique applied for 4 weeks did not show a significant change in erector spinae muscle stiffness in individuals with chronic low back pain [26]. In addition to the 4-week period, we anticipate that other factors such as range of motion may also affect muscle viscoelastic properties. This is because one of the main effects of increased range of motion is usually decreased muscle stiffness [26]. Although this was not evaluated in our study, there were no deficits or limitations of joint range of motion in the healthy individuals. Therefore, the unchanged viscoelastic properties may also be due to the absence of any change in range of motion. In conclusion, it is thought that more research is needed on the effects of the Pilates method on the tonus and stiffness levels in superficial muscles.

Little information is available on the muscle mechanical properties of the lumbar spine in adults with low back pain [5]. In one of the limited number of studies, it was reported that individuals suffering from low back pain may have differences in muscle tone and stiffness in the spinal region compared to the healthy control group [1]. On the other hand, although no statistical analysis was performed in our study, healthy individuals and individuals with chronic low back pain were similar in terms of initial muscle viscoelastic values (no more than 5% difference). We attribute the lack of significant difference in the final values of both groups to the fact that the baseline values were close to each other and Pilates-based exercises had no effect on muscle viscoelastic values.

The effects of Pilates on the mat in individuals with chronic low back pain have been examined in many studies [9,29]. In our study, differently, the effects of reformer Pilates exercises in individuals with chronic low back pain were examined. The reason for choosing reformer Pilates in individuals with chronic low back pain is that this method contributes to posture alignment and lumbopelvic stability as well as exercising the extremities. Pilates exercises can be performed on a mat or with the help of special equipment such as the reformer machine, Trapeze Table/Cadillac, Ladder Barrel, and Step/Wunda Chair. The reformer machine is composed of a platform integrated within a wooden or metal frame. This apparatus is connected to a system of springs, pulleys, and ropes [28]. Another important finding of our study was that reformer Pilates-based exercises decreased pain, disability, and kinesiophobia levels and increased quality of life and sleep in individuals with chronic low back pain. On the other hand, the effect of Pilates-based exercises on healthy individuals was significant only in terms of fear of movement/reinjury. Pilates exercises are known to reduce pain and improve postural release in adults with chronic low back pain [30]. Previous evidence has shown that Pilates-based exercise approaches improve aerobic fitness and quality of life, strengthen the lower limb muscles, and improve postural stability, balance, and fall risk [8]. Pilates exercises have been shown to be one of the most recommended exercise methods due to improvements in function, disability, and pain in adults with chronic low back pain [31]. In the systematic review by Romao et al. that examined the effect of Pilates exercises in individuals with chronic low back pain, it was stated that Pilates exercises are an excellent option to promote health and help prevent low back pain [32]. The results of our study are in parallel with the literature. We think that the main reason for this situation is that Pilates exercises strengthen the core muscles and alleviate pain, and the Pilates method is easy to apply and accepted easily by adults because it is a popular method, and as a result, positive effects towards recovery increase.

Another finding of our study was that changes in muscle viscoelastic properties were not affected by changes in pain and disability levels or sleep and quality of life. We make this finding as a natural inference. This is because these parameters changed with Pilates-based exercises in individuals with chronic pain, but not in the healthy control group. Although there was a change in these parameters in one group but not in the other, there were no significant changes in the erector spinae and rectus abdominis muscle tone and stiffness levels in both groups after performing Pilates-based exercises. Indeed, Ambrose Lo et al. did not find a significant relationship between pain intensity and mechanical properties of paraspinal muscles in their study [5]. In conclusion, as a result of the findings obtained in our study, we conclude that abdominal and lumbar muscle tone and stiffness may not always be associated with underlying pathologies or changes in symptoms.

The last important finding obtained in our study is that Pilates-based exercises cause regional thinning such as to the chest, waist, hips, and thighs. Nowadays, Pilates is a popular therapeutic exercise method among individuals of all ages and genders. One of the main reasons for this is probably the effect of Pilates exercises on anthropometric measurements such as body fat reduction [29]. Indeed, the majority of the scientific literature has focused on the body composition and anthropometric values of Pilates exercises [29]. Some studies have shown that Pilates exercises provide significant changes in measurements such as waist circumference and waist-to-height ratio [33] or body mass index, waist/hip, or waist/height [34]. Lee et al. showed that significant reductions in waist circumference measurements occurred after Pilates exercises were performed for 4 weeks [35].

As a result, finding significant thinning in anthropometric values was a result we expected, even though the duration of Pilates was relatively short. Our reason for performing anthropometric measurements in this study was to observe whether there was a change in muscle viscoelastic properties due to regional thinning. Indeed, although regional thinning occurred, no significant changes were observed in the tonus and stiffness values of the erector spinae and abdominal muscles. Based on this finding, we can state that muscle viscoelastic properties may not change directly after regional thinning.

### Limitations

There are several limitations in our study. The first of these is that although all of the individuals with chronic low back pain included in our study were women, there was a small number of men among the healthy individuals in the control group, despite a large female population. With regard to gender imbalance, we report that the menstrual cycle has the potential to affect viscoelastic measurements in women, and this should therefore be taken into consideration. Furthermore, the lack of monitoring of individuals’ daily fluid intake is a significant limitation. Another important limitation was the relatively short duration of Pilates practice in this study due to the requirements of the clinical center. Significant regional thinning occurred in individuals in both groups following Pilates exercises. However, as weight measurements were not taken after four weeks, it is unclear whether this thinning was due to muscle loss, fat loss, oedema, etc. This may be another limitation. In our study, we used questionnaires such as the Kinesiophobia Scale and the Oswestry Disability Index, which are usually employed with disease-based populations, with both individuals with and without chronic low back pain. This should be noted as a significant limitation. Lastly, we evaluated the viscoelastic properties of only superficial muscles using the Myoton device. Therefore, the effects of Pilates cannot be generalized to all muscle groups.

## 5. Conclusions

In conclusion, performing Pilates exercises for four weeks did not result in significant changes in muscle tone and stiffness of the main core muscles, such as the erector spinae and rectus abdominis, in the lumbar and abdominal regions in either healthy individuals or those with chronic low back pain. The presence of chronic low back pain did not appear to affect the effects of performing Pilates exercises for 4 weeks on the viscoelastic properties of the erector spinae and rectus abdominis muscles. The effectiveness of Pilates exercises in reducing pain, disability, and kinesiophobia, providing regional thinning in some areas, and improving sleep and quality of life has been proven in individuals with chronic low back pain.

## Figures and Tables

**Table 1:** Descriptive statistics of the demographic status of participants.

Variables	CG Subjects (*n* = 24)	CLBP Subjects (*n* = 28)	*p*
Age (years)	33.20 ± 10.43	34.50 ± 12.07	0.727
Height (cm)	170.37 ± 8.47	165.67 ± 6.13	0.049 *
Weight (kg)	64.88 ± 16.51	59.78 ± 11.40	0.468
BMI (kg/m^2^)	22.09 ± 3.98	21.71 ± 3.54	0.854
Gender	18 Female/6 Male	28 Female	-

BMI: body mass index; CLBP: chronic low back pain; CG: control group; * *p* < 0.05.

**Table 2:** Comparison of the muscle viscoelastic values in both groups.

	Subjects with	Baseline	Follow-Up	*p* ^1^	Δ (4th Week—Baseline)	*p* ^2^
Rectus Abdominis Tone (Hz)	CLBP (*n* = 28)	13.64 ± 2.93	13.24 ± 2.53	0.594	−0.18 ± 1.70	0.322
	CG (*n* = 24)	13.92 ± 1.84	13.74 ± 1.47	0.345	−0.18 ± 0.78	
Rectus Abdominis Stiffness (N/m)	CLBP (*n* = 28)	223.57 ± 61.47	213.00 ± 60.99	0.946	−5.52 ± 48.88	0.435
	CG (*n* = 24)	233.00 ± 29.16	222.83 ± 31.83	0.247	−10.16 ± 32.23	
Erector Spinae Tone (Hz)	CLBP (*n* = 28)	15.57 ± 2.07	15.51 ± 2.34	0.885	−0.06 ± 1.13	0.734
	CG (*n* = 24)	15.63 ± 2.89	15.57 ± 2.37	0.845	−0.05 ± 1.56	
Erector Spinae Stiffness (N/m)	CLBP (*n* = 28)	275.75 ± 62.01	266.92 ± 62.47	0.509	−8.82 ± 33.25	0.949
	CG (*n* = 24)	285.66 ± 74.64	277.91 ± 63.58	0.407	−7.75 ± 35.27	

*p* ^1^ < 0.05: significant intragroup difference; *p* ^2^ < 0.05: significant intergroup difference; CLBP: chronic low back pain; CG: control group.

**Table 3:** Comparison of the health-related outcomes in both groups.

Variables	Subjects with	Baseline Mean ± SD	Follow-Up Mean ± SD	*p* ^1^	Δ (4th Week—Baseline)	*p* ^2^
Pain Intensity (cm)	CLBP (*n* = 28)	5.42 ± 1.73	3.71 ± 1.51	<0.001 *	−1.71 ± 1.11	<0.001 *
	CG (*n* = 24)	00.00 ± 00.00	00.00 ± 00.00	1.000	0.00 ± 0.00	
Sleep Quality (score)	CLBP (*n* = 28)	5.64 ± 3.46	4.92 ± 2.87	<0.001 *	−0.71 ± 1.11	0.042 *
	CG (*n* = 24)	2.95 ± 2.23	2.95 ± 1.98	0.971	0.00 ± 0.97	
Quality of Life (score)	CLBP (*n* = 28)	58.73 ± 70.25	49.85 ± 65.82	0.019 *	−8.88 ± 28.03	0.356
	CG (*n* = 24)	21.12 ± 22.42	15.93 ± 19.59	0.055	−5.18 ± 19.24	
Kinesiophobia (score)	CLBP (*n* = 28)	31.10 ± 8.41	26.10 ± 7.45	<0.001 *	−5.00 ± 3.51	0.126
	CG (*n* = 24)	29.00 ± 6.91	25.87 ± 6.27	<0.001 *	−3.12 ± 3.84	
Disability Level (score)	CLBP (*n* = 28)	6.21 ± 5.28	4.50 ± 4.54	0.003 *	−1.71 ± 2.70	0.225
	CG (*n* = 24)	2.50 ± 3.83	2.08 ± 3.46	0.064	−0.41 ± 2.94	
Chest (cm)	CLBP (*n* = 28)	88.32 ± 8.33	87.33 ± 7.98	0.002 *	−0.98 ± 1.47	0.824
	CG (*n* = 24)	90.14 ± 11.67	89.10 ± 10.65	0.031 *	−1.04 ± 2.07	
Waist (cm)	CLBP (*n* = 28)	73.64 ± 6.71	72.33 ± 6.86	0.015 *	−1.30 ± 2.67	0.682
	CG (*n* = 24)	75.75 ± 13.57	74.83 ± 13.43	0.047 *	−0.91 ± 1.95	
Abdomen (cm)	CLBP (*n* = 28)	74.23 ± 9.40	73.14 ± 9.22	0.091	−1.08 ± 3.26	0.747
	CG (*n* = 24)	77.12 ± 14.43	76.52 ± 14.16	0.109	−0.60 ± 2.31	
Hip (cm)	CLBP (*n* = 28)	98.19 ± 9.84	97.42 ± 9.20	0.016 *	−0.76 ± 1.44	0.381
	CG (*n* = 24)	98.08 ± 9.41	96.97 ± 8.57	0.022 *	−1.10 ± 2.44	
Thigh (cm)	CLBP (*n* = 28)	54.23 ± 4.65	53.83 ± 4.65	0.012 *	−0.39 ± 0.80	0.320
	CG (*n* = 24)	54.16 ± 4.78	53.60 ± 4.83	0.003 *	−0.56 ± 0.72	

*p* ^1^ < 0.05: significant intragroup difference; *p* ^2^ < 0.05: significant intergroup difference; CLBP: chronic low back pain; CG: control group; * *p* < 0.05.

## Data Availability

All data generated or analyzed during this study are included in this published article.

## Supplementary Materials

The following supporting information can be downloaded at: https://www.mdpi.com/article/10.3390/healthcare14040448/s1, Supplementary Material S1: List of Pilates exercises.
