# Supplementary material for: Pilates-Based Training and Its Influence on Muscle Viscoelasticity and Health-Related Outcomes in Chronic Low Back Pain: A Comparative Study
Source: Healthcare (Basel). 2026 Feb 11;14(4):448. doi: 10.3390/healthcare14040448 (PMC12940347; doi:10.3390/healthcare14040448)
Supplement: Supplementary file 1 [file healthcare-14-00448-s001.zip › healthcare-4101473-supplementary.pdf]

Supplement S1. Pilates Exercise List

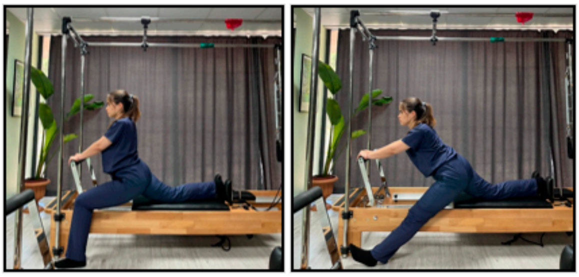

Standing Lunge

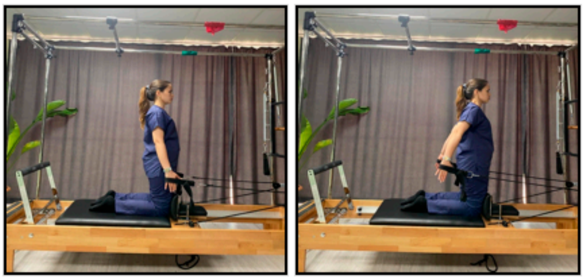

Thoracic Expansion

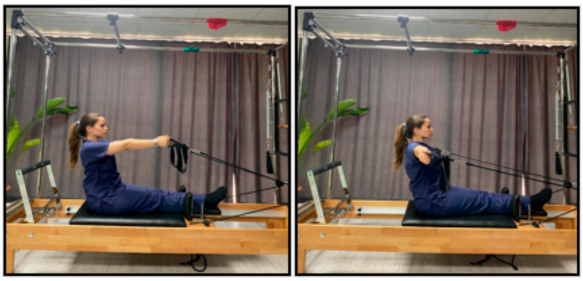

Hug a Tree

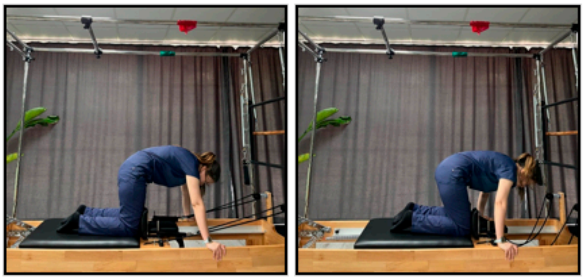

Reverse Quadripedal

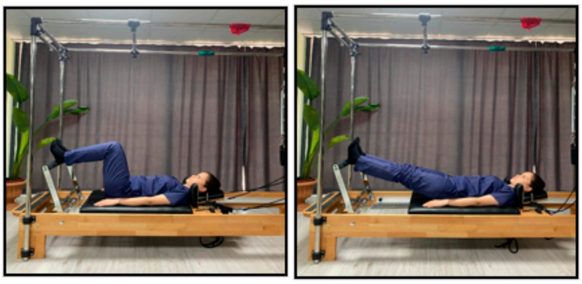

Footwork Series

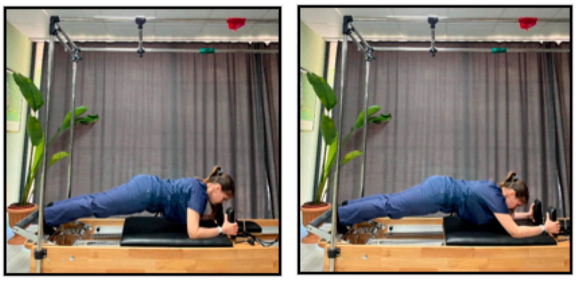

Balance Exercises

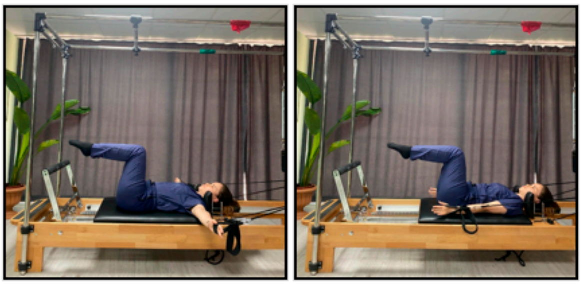

Arm Adduction

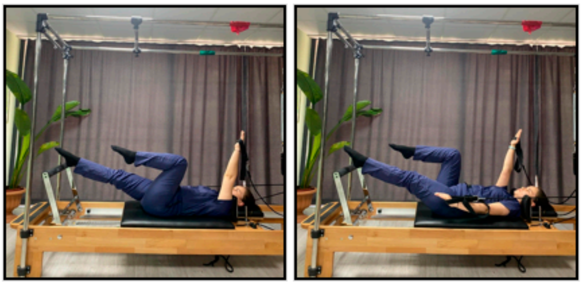

Coordination

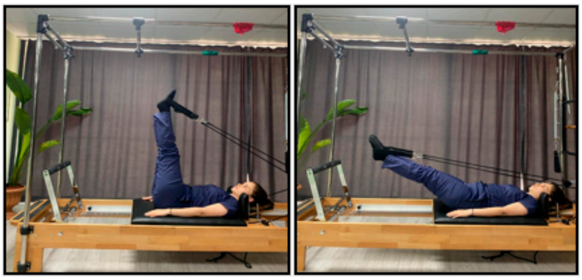

Hip Extension

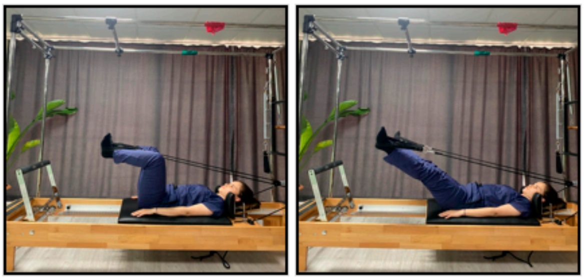

Frog Exercises

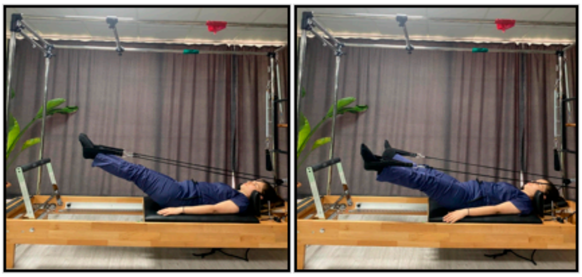

Circle Exercises

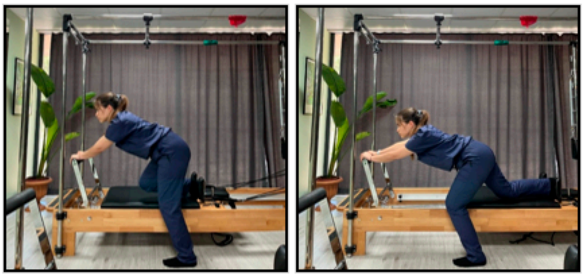

Scooter

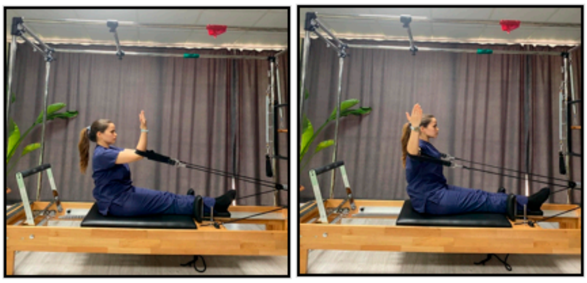

Rhomboid Strengthening

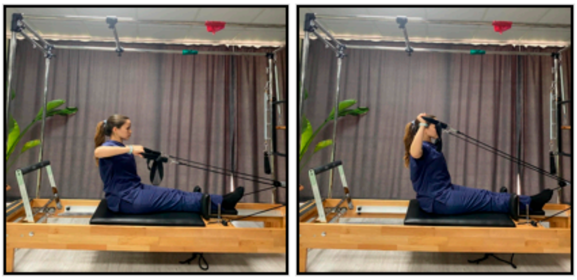

Rotator Cuff

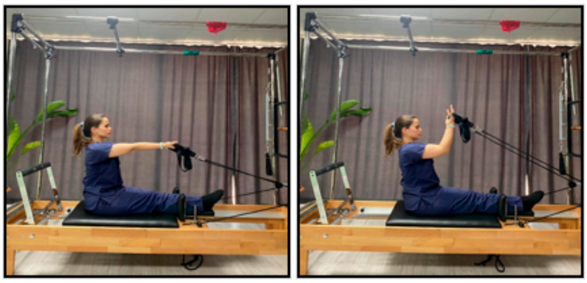

Biceps Muscle

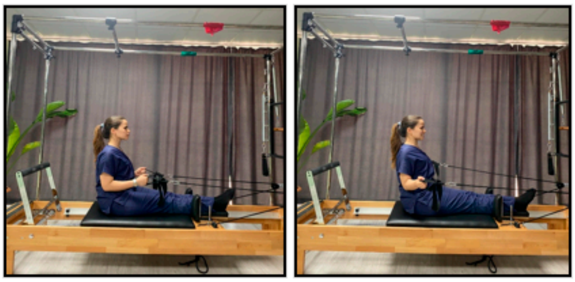

External Rotation

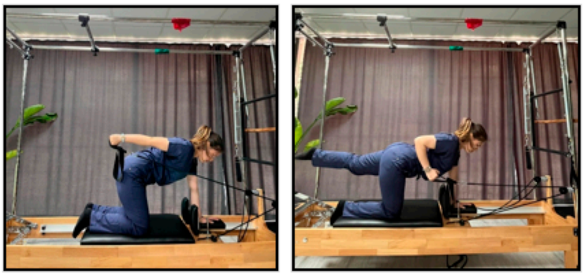

Quadripedal Kickback

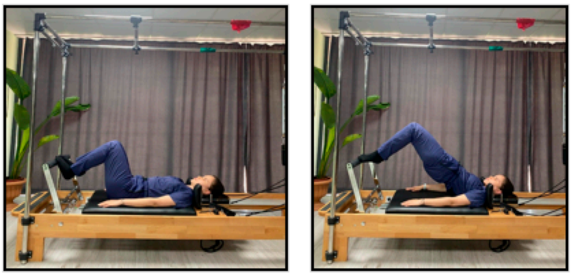

Bottom Lift

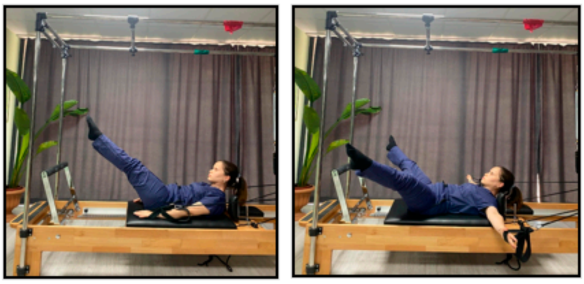

Abd Openings

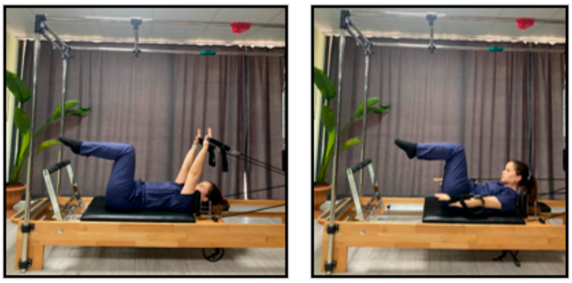

Hundred Prep

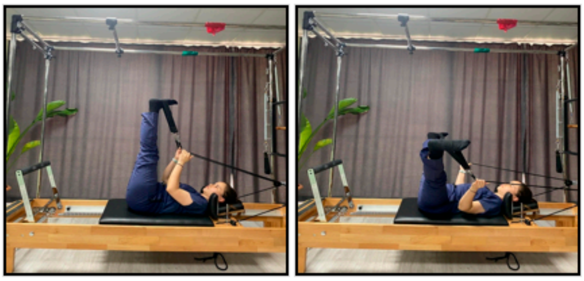

Stretching Exercises

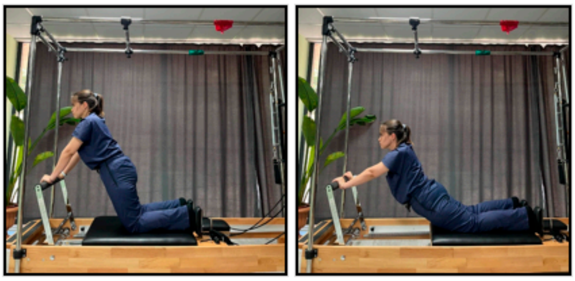

Stretching Exercises

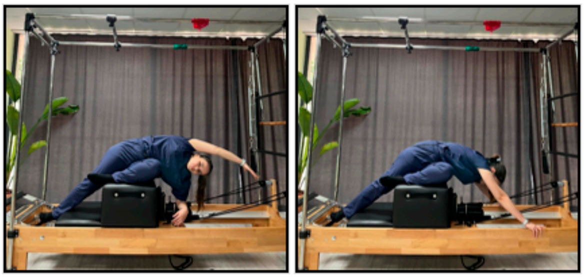

Stretching Exercises

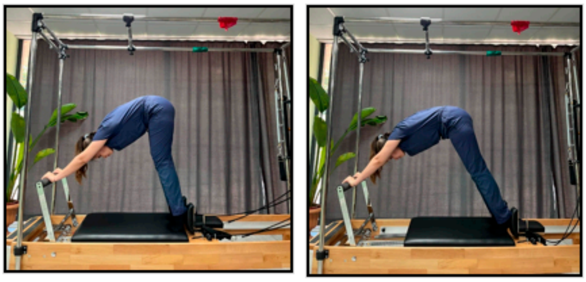

Stretching Exercises
